# Clinical and prognostic significance of serum transforming growth factor-beta1 levels in patients with pancreatic ductal adenocarcinoma

**DOI:** 10.1590/1414-431X20165485

**Published:** 2016-07-25

**Authors:** J. Zhao, Y. Liang, Q. Yin, S. Liu, Q. Wang, Y. Tang, C. Cao

**Affiliations:** 1 Department of Clinical Laboratory, People's Hospital of Weifang, Shandong, China; 2 Department of Surgery, People's Hospital of Rizhao, Shandong, China; 3 Department of Oncology, People's Hospital of Rizhao, Shandong, China; 4 Department of Clinical Laboratory, People's Hospital of Zhangqiu, Shandong, China; 5 Department of Medical Laboratory Diagnosis Center, Jinan Central Hospital, Shandong, China

**Keywords:** Pancreatic ductal adenocarcinoma, Marker, TGF-β1

## Abstract

Pancreatic ductal adenocarcinoma (PDAC) has a poor 5-year survival rate of 5%. Biomarkers for the early detection of pancreatic cancer are urgently needed. Transforming growth factor-beta1 (TGF-β1) is elevated in the tissues and plasma of patients with PDAC. However, no studies systemically report prognostic significance of plasma TGF-β1 levels in PDAC. In the present study, we assessed the prognostic significance of serum TGF-β levels in patients with PDAC. TGF-β levels were determined in serum from 146 PDAC patients, and 58 patients with benign pancreatic conditions. Regression models were used to correlate TGF-β levels to gender, age, stage, class, and metastasis. Survival analyses were performed using multivariate Cox models. Serum levels of TGF-β1 distinguished PDAC from benign pancreatic conditions (P<0.001) and healthy control subjects (P<0.001). Serum levels of TGF-β also distinguished tumor stage (P=0.002) and lymph node metastasis (P=0.001). High serum levels of TGF-β1 were significantly correlated with reduced patient survival. Multivariate analysis revealed that TGF-β1, lymph node metastasis and tumor stage were independent factors for PDAC survival. Our results indicate that serum TGF-β1 may be used as a potential prognostic marker for PDAC.

## Introduction

Pancreatic ductal adenocarcinoma (PDAC) is one of the most fatal cancers, with a 5-year survival of less than 5% due to its high recurrence rate, despite the multimodality treatments (1). Surgical resection is the only chance of cure, but due to advanced stage at presentation only 20% of patients have resectable tumors (2). Of these, only 15% will have early-stage cancers (3). When resection is possible and followed by adjuvant therapy, the 5-year survival is higher, at 20–30% (4). It is clear that early detection of smaller tumors is necessary to improve resectability rates and survival.

CA 19-9 is currently the gold standard serum biomarker for the diagnosis of PDAC. Recent evidence has supported the utility of CA 19-9 as a prognostic biomarker for PDAC, especially with regards to predicting survival following clinical treatment (5). CEA is likely the second most used biomarker for PDAC. However, neither of the two biomarkers possesses the accuracy desirable for screening asymptomatic populations (6). The past decade has witnessed intensive study and impressive progress in searching for novel biomarkers, but further studies are needed to find more sensitive, specific, and cost-effective biomarkers for diagnosis and prognosis.

Transforming growth factor (TGF)-β is involved in physiological processes, such as wound healing, tissue development, and remodeling. TGF-β has also been implicated in many pathological conditions, including cancer, and has been shown to regulate a number of events such as angiogenesis, immune suppression, and cell migration (7,8). Clinically, TGF-β1 is often elevated in the plasma of patients with esophageal cancer (9), gastric cancer (10), osteosarcoma (11), melanoma (12), and increased serum TGF-β1 levels is related with poor prognosis (13,14).

Multiple experimental studies have shown correlations between TGF-β1 expression and increased tumorigenicity and increased invasion in pancreatic cancer cells (15). In pancreatic cancer, expression of TGF-β1 in the primary tumor can predict survival of patients undergoing surgical resection (16). However, its clinicopathological and prognostic significances in serum of patients with PDAC remain unknown.

In the present study, we evaluated the levels of TGF-β1 in the blood of PDAC patients and its clinical and/or prognostic significance.

## Material and Methods

The People's Hospital of Weifang approved the retrospective use of the data for the study. Data were acquired retrospectively from electronic medical records.

### Patients

Pretreatment serum samples were obtained from 146 patients with histologically or cytologically confirmed PDAC, from January 2006 to December 2010. Serum samples from 58 patients with chronic pancreatitis, benign pancreatic cysts, or other benign pancreatic neoplasms were also analyzed. The variables evaluated included age, gender, tumor size, differentiation status, lymph node involvement and TNM stage. None of the patients with benign pancreatic diseases or the healthy controls (selected from the Center of Physical Examination of People's Hospital of Weifang and Central Hospital of Jinan) had a history of malignancies. Serum from 204 subjects was analyzed in this study (Table 1).

**Table:**
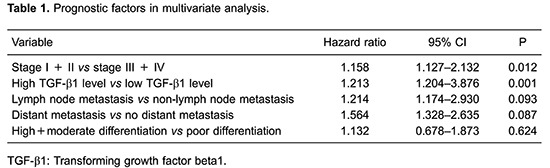

### Blood acquisition and serum preparation

A total of 5-mL venous blood was collected in a sterile 15 mL plastic Falcon tube, left to clot and then centrifuged at 11,000 *g* for 15 min. Serum samples were stored at –80°C until required for the assay.

### Detection of TGF-β1 expression in serum

Quantitative enzyme-linked immunosorbent assay (ELISA) kits were used to assess the levels of human TGF-β1 according to the manufacturer's instructions. A total of 200 µL of prediluted sera was added to micro-titer wells precoated with anti-human TGF-β1 polyclonal antibodies followed by a biotin-conjugated mouse anti-TGF-β1 antibodies and streptavidin-horseradish peroxidase. Color was developed using a tetramethyl benzidine-hydrogen dioxide mixture and terminated with sulfuric acid. The absorbance of each well was determined using a spectrophotometer.

### Statistical analysis

Data are reported as means±SD. The associations between TGF-β1 level and clinicopathological variables were examined using the χ^2^ test. The association with survival was analyzed using Kaplan-Meier analysis and curves were compared using the log-rank test and Cox regression analysis to adjust for other prognostic indicators. The receiver operating characteristic (ROC) curve was determined and the area under the curve (AUC) was calculated as a comparative measure of diagnostic accuracy. Multivariate Cox regression analysis was used to analyze the relationship between independent and dependent variables. All statistical analyses were performed with the IBM SPSS Statistical software (SPSS Statistics 17.0, USA). Statistical significance was defined as P*<*0.05.

## Results

### TGF-β1 in the sera of patients and controls

Serum TGF-β1 levels were 57.6±23.2 ng/mL (mean±SD) in healthy control subjects, 64.5±27.4 ng/mL in benign pancreatic conditions, and 237.6±45.3 ng/mL in PDAC patients. There was a statistically significant difference between TGF-β1 serum levels in patients with PDAC and control groups (P<0.001), but levels were not elevated in patients with benign pancreatic conditions compared to healthy control subjects (P=0.437).

### TGF-β1 levels and association with clinicopathological characteristics in PDAC

We used the 57.6 ng/mL TGF-β1 level of the healthy controls as the cut-off value. Values >57.6 ng/mL were defined as high, and ≤ 57.6 ng/mL as low. In PDAC patients, 72.6% (106/146) had high TGF-β1 levels, and 27.4% (40/146) had low levels. Serum TGF-β1 levels were significantly related with tumor stage (P=0.001) and lymph node metastasis (P=0.001). There was no significant correlation between TGF-β1 and other factors, such as age, gender, histological differentiation, and distant metastasis.

### Sensitivity and specificity of TGFβ1 levels in the diagnosis of PDAC

**Figure 1:**
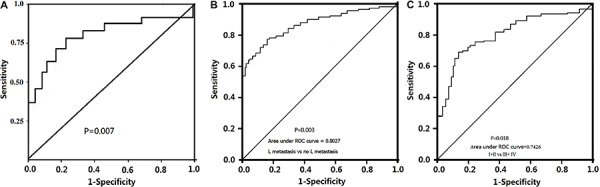
Receiver operating characteristic curve analysis of serum TGF-β1 in pancreatic ductal adenocarcinoma patients *vs* benign pancreas disease (*A*), for lymph node metastasis *vs* non-lymph node metastasis (*B*), and for stage I+II *vs* stage III+IV (*C*).

ROC curves were generated for serum TGFβ1 to evaluate its ability to distinguish PDTC from benign pancreas disease, lymph node metastasis from non-lymph node metastasis and stages I and II from stages III and IV. Our results suggest that serum TGF-β1 is able to distinguish PDTC from benign pancreas disease with an AUC of 0.794, specificity of 76.4% and sensitivity of 83% (P=0.007, Figure 1A). Serum TGF-β1 is able to distinguish lymph node metastasis from non-lymph node metastasis with an AUC of 0.8027, specificity of 80.4% and sensitivity of 75.6% (P=0.003, Figure 1B). In addition, serum TGF-β1 distinguished stages I and II from stage III and IV with a AUC of 0.7426, specificities of 68% and sensitivity of 72% (P=0.0018, Figure 1C).

### Influence of TGF-β1 level on overall survival in PDAC

**Figure 2:**
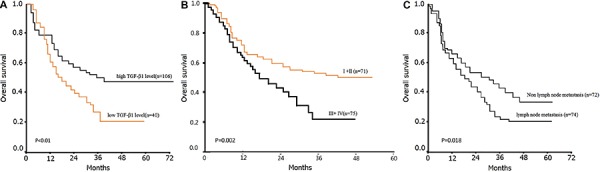
Kaplan Meier survival curve for low and high TGF-β1 levels in plasma of pancreatic ductal adenocarcinoma patients (*A*), for patients with stage I and II or stage III and IV (*B*), and for pancreatic cancer patients with lymph node metastasis and non-lymph node metastasis (*C*).

Median survival from the time of PDAC operation for all patients was 18 months (range of 0.6–26 months), with a 5-year overall survival of 18.7%. By univariate analysis, the 5-year median overall survival was 21.7% in low TGF-β1 level groups, which was significantly higher than the high TGF-β1 level groups (15.3%; P<0.01, Figure 2A).

Cox regression multivariate analysis indicated that only serum TGF-β1 level (P=0.001; HR=1.213, 95%CI: 1.204–3.876), tumor stage of PDAC (P=0.012; HR=1.158, 95%CI: 1.127–2.132) and lymph node metastasis (P=0.018; HR=1.214, 95%CI: 1.374–2.93) were independent prognostic factors for patients with PDAC (Table 1). Kaplan-Meier survival curves for stage and lymph node metastasis of PDAC are shown in Figure 2 B and C).

## Discussion

TGF-β1 is a pleiotropic cytokine that, depending on the cell niche, can display either anti-inflammatory or proinflammatory effects (17). A recent study has found that TGF-β1 could shape the metastatic niche and favor the maintenance of an immunosuppressive phenotype in mesenchymal cells (18–20). TGF-β has a dual role in tumor development including tumor suppression through inhibition of proliferation and induction of apoptosis in multiple cell types, or promotion of tumor cell invasiveness and metastasis through modulation of the immune system as well as of the tumor microenvironment (21). Many studies have found that TGF-β1 is activated and released into the blood under ischemia and hypoxia stress, and it is markedly induced and rapidly activated in the infarcted myocardium. Bioactive TGF-β1 is released in the cardiac extracellular fluids 3–5 h following reperfused infarction (22). Tissue and serum TGF-β1 were also significantly increased in patients with cancers, indicating its predictive and prognostic roles in patients with this disease.

In our study, serum levels of TGF-β1 were found to be significantly higher in PDAC patients than in normal controls or patients with benign pancreas disease. Furthermore, the serum TGF-β1 level increased with increased tumor stage, lymph node metastasis and distant metastasis. These findings are supported by Lin et al. (10,11), who stated that TGF-β1 levels reflected tumor stage and metastasis.

Recent investigations of patients with PDAC have shown significantly elevated serum levels of TGF-β1, which could be well correlated with the risk of death (23). In this study, data clearly showed that patients with elevated TGF-β1 in the serum had a shorter survival. Furthermore, TGF-β1 >57.6 ng/mL had a sensitivity of 83% and a specificity of 76.4% for detecting individuals with PDAC. In addition, multivariate analysis showed that TGF-β1 is an independent prognostic factor. We believe that TGF-β1 may be considered to be a biomarker for predicting the probability of PDAC in the future. We also found in our study that TGF-β1 levels are predictive of metastasis. We found that high baseline TGF-β1 levels were the best predictor of PDAC metastasis. TGF-β1 levels were significantly higher in patients with metastasis compared to those without it. Furthermore, TGF-β1 could also distinguish stage I and II from stage III and IV.

In conclusion, we found that patients with high TGF-β1 plasma levels had an increased risk of metastasis and advanced PDAC. In addition, the high levels were associated with short survival and poor prognosis of PDAC. Our results indicate that serum TGF-β may be used as a potential prognostic marker for the PDAC.
